# The Use of Putative Dialysis Initiation Time in Comparative Outcomes of Patients with Advanced Chronic Kidney Disease: Methodological Aspects

**DOI:** 10.6000/1929-6029.2022.11.16

**Published:** 2022

**Authors:** Danh V. Nguyen, Esra Kurum, Damla Senturk

**Affiliations:** 1 Department of Medicine, University of California Irvine, Orange, CA 92868, USA; 2 Department of Statistics, University of California, Riverside, CA 92521, USA; 3 Department of Biostatistics, University of California, Los Angeles, CA 90095, USA

**Keywords:** Chronic kidney disease, end-stage kidney disease, dialysis, linear mixed effects model, survival, propensity score

## Abstract

The latest data from the United States Renal Data Systems show over 134,000 individuals with end-stage kidney disease (ESKD) starting dialysis in the year 2019. ESKD patients on dialysis, the default treatment strategy, have high mortality and hospitalization, especially in the first year of dialysis. An alternative treatment strategy is (non-dialysis) conservative management (CM). The relative effectiveness of CM with respect to various patient outcomes, including survival, hospitalization, and health-related quality of life among others, especially in elderly ESKD or advanced chronic kidney disease patients with serious comorbidities, is an active area of research. A technical challenge inherent in comparing patient outcomes between CM and dialysis patient groups is that the start of follow-up time is “not defined” for patients on CM because they do not initiate dialysis. One solution is the use of putative dialysis initiation (PDI) time. In this work, we examine the validity of the use of PDI time to determine the start of follow-up for longitudinal retrospective and prospective cohort studies involving CM. We propose and assess the efficacy of estimating PDI time using linear mixed effects model of kidney function decline over time via simulation studies. We also illustrate how the estimated PDI time can be used to effectively estimate the survival distribution.

## 1. INTRODUCTION

In the United States, nearly 15% or 34 million adults have chronic kidney disease (CKD) as of 2021[1–2] and each year since 2014 over 120,000 individuals transition to dialysis [3]. The latest data from the United States Renal Data Systems (USRDS) for the year 2019 shows over 134,000 individuals transitioned to dialysis [1]. In the US (as well as other nations) dialysis is a default treatment strategy, made possible by the 1972 End-Stage Renal Diseases (ESRD) legislation, extending Medicare benefits for all patients on dialysis to prolong life. Although dialysis is the default treatment, its benefit with respect to important patient outcomes, including survival, hospitalization and readmission, and health-related quality of life (HRQOL) outcomes, particularly for older patients (e.g., age ≥ 70) with serious comorbidities, may not be optimal. Thus, multidisciplinary support for patients with end-stage kidney disease (ESKD) who choose not to initiate dialysis, is an alternative treatment strategy called “conservative management” (CM), especially among elderly patients with serious comorbidities [4]. This alternative treatment strategy to dialysis is also variously called “maximum conservative management,” “palliative renal care,” and non-dialysis treatment [5]. Studies have documented that patients on dialysis have high mortality rate in the first year [3, 6–8], frequent hospitalization and readmission [9–12], low HRQOL [13–14], decline in physical functioning [15–18], and high cost. For the older ESKD population with major comorbidities, patients starting dialysis may not have a survival benefit compared with patients choosing CM [19–24], although the risk of hospitalization and readmission is higher [9–12].

When evaluating patient outcomes (including survival, hospitalization, HRQOL, etc.) after “initiation of dialysis” between patients on CM treatment versus patients who initiate dialysis in both prospective and retrospective longitudinal studies, there is a fundamental technical issue of *undefined* (*ambiguous*) *follow-up time for patients on CM* because they *do not start* dialysis. For dialysis patients, the follow-up time is unambiguous since it is the time when they transitioned to dialysis; thus, the focus of this paper is on estimating the follow-up (time at risk) for patients on CM. For instance, to be concrete, in order to compare survival or hospitalization rate during the first year (after “initiating” dialysis), *the start of follow-up time* to assess survival (or hospitalization) for patients on CM must be defined since they do not start dialysis. One possible solution is to consider the question, “If a patient on CM was to start dialysis, when would that likely have occurred for them?” Thus, one practical approach is to assume that the *putative* start of dialysis for a patient on CM is the time when their kidney function level, based on estimated glomerular filtration rate (eGFR ml/min/1.73m^2^), is comparable (equal) to the average level of kidney function among patients who started dialysis treatment [10]. The putative dialysis initiation (PDI) time can be estimated based on the longitudinal eGFR trajectory for *each* patient in the CM group and was implemented in practice using subject-specific simple linear regression [10], which can be unstable due to the small number of repeated eGFR measurements per subject.

A systematic assessment of the validity of the use of PDI time for patients on CM have not been considered to date; thus, in this paper, we consider this issue and using simulation studies, we illustrate the inefficiency of using subject-specific linear regression to estimate PDI times and suggest a more stable approach to using subject-specific predictions from linear mixed effects model to estimate PDI times for patients in the CM group. We illustrate the approach to estimate the survival distribution for CM patients using the estimated putative survival times. Although we illustrate the method with a survival outcome, the use of PDI times is applicable to comparative analysis of all of the aforementioned outcomes (survival, hospitalization, readmission, HRQOL, physical/mental functioning, health care utilization, cost etc.).

The remainder of our paper is organized as follows. In Section 2, we describe the estimation of PDI time and a simulation study design to assess the efficacy of PDI estimation. Results are reported in Section 3 and we conclude with a discussion in Section 4.

## 2. METHODS

### 2.1. Estimation of PDI Time

**Figure 1:**
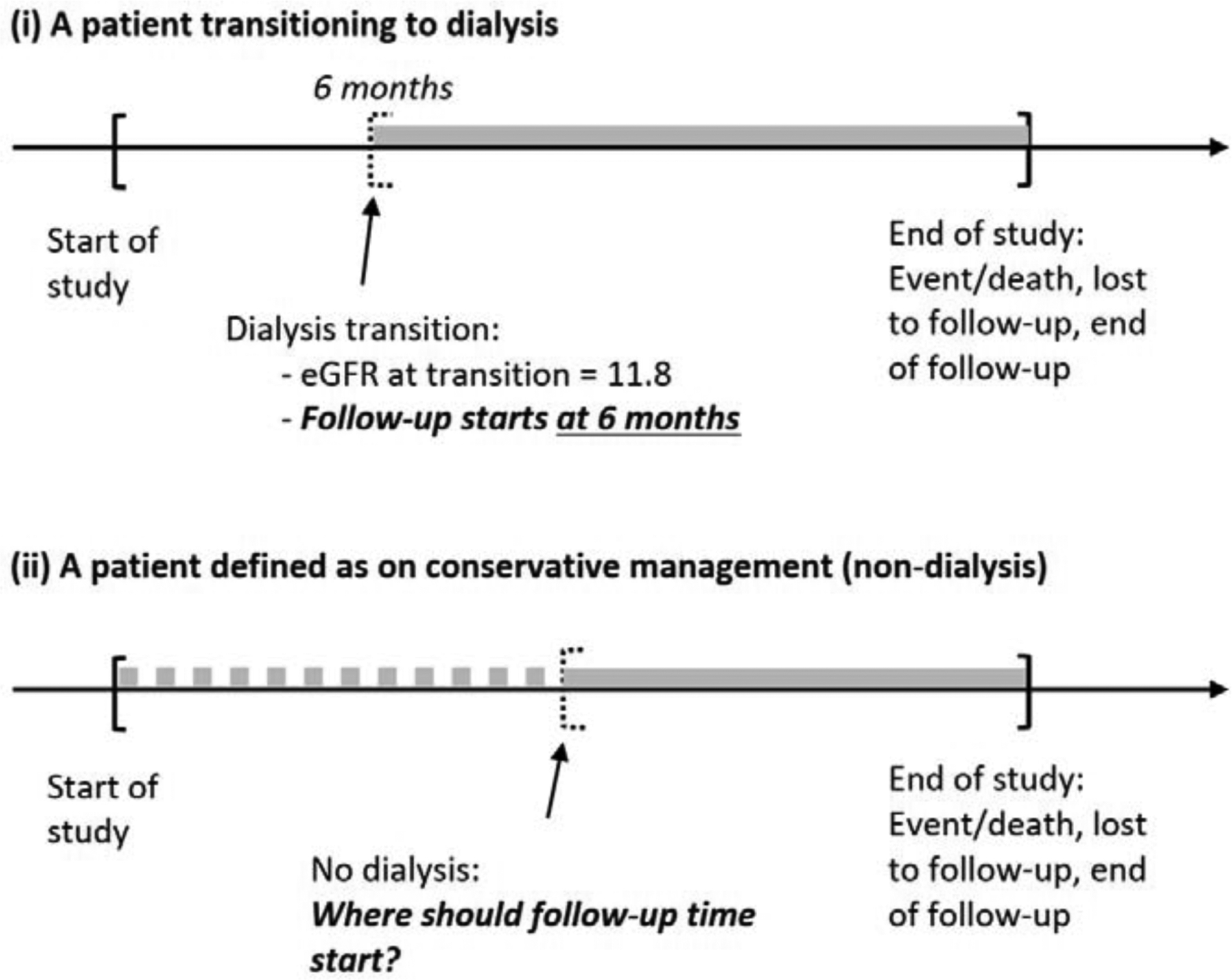
Illustration of typical follow-up time for an advanced CKD patient (i) initiating dialysis versus (ii) conservative management (non-dialysis). “Start of study” is an arbitrary designation of when the “data collection” starts which could be the date/time at which a CKD patient has eGFR ≤ 25 (i.e., “advanced CKD” in a database) in a retrospective cohort study or the study entry date for a prospective cohort study of advanced CKD patients (where, for instance, all patients with eGFR ≤ 25 are eligible). The “start of the study” and the subsequent time of the “start of follow-up” as depicted should not be confused with left-censored data.

For both retrospective and prospective longitudinal studies, the follow-up time period (start and end of follow-up) must be well-defined with respect to a patient outcome for comparing outcomes, such as survival, among treatment groups. As introduced in the previous section, this is challenging for comparing CM to dialysis treatment groups in advanced CKD patients due to the fact that patients in the CM group do not start dialysis. Figure 1 illustrate the typical follow-up time period for assessment of survival for: (i) a patient on dialysis where the start of follow-up is known at 6 months after the study start; (ii) a patient on CM where a decision on the start of follow-up is required in order to compare survival.

**Figure 2:**
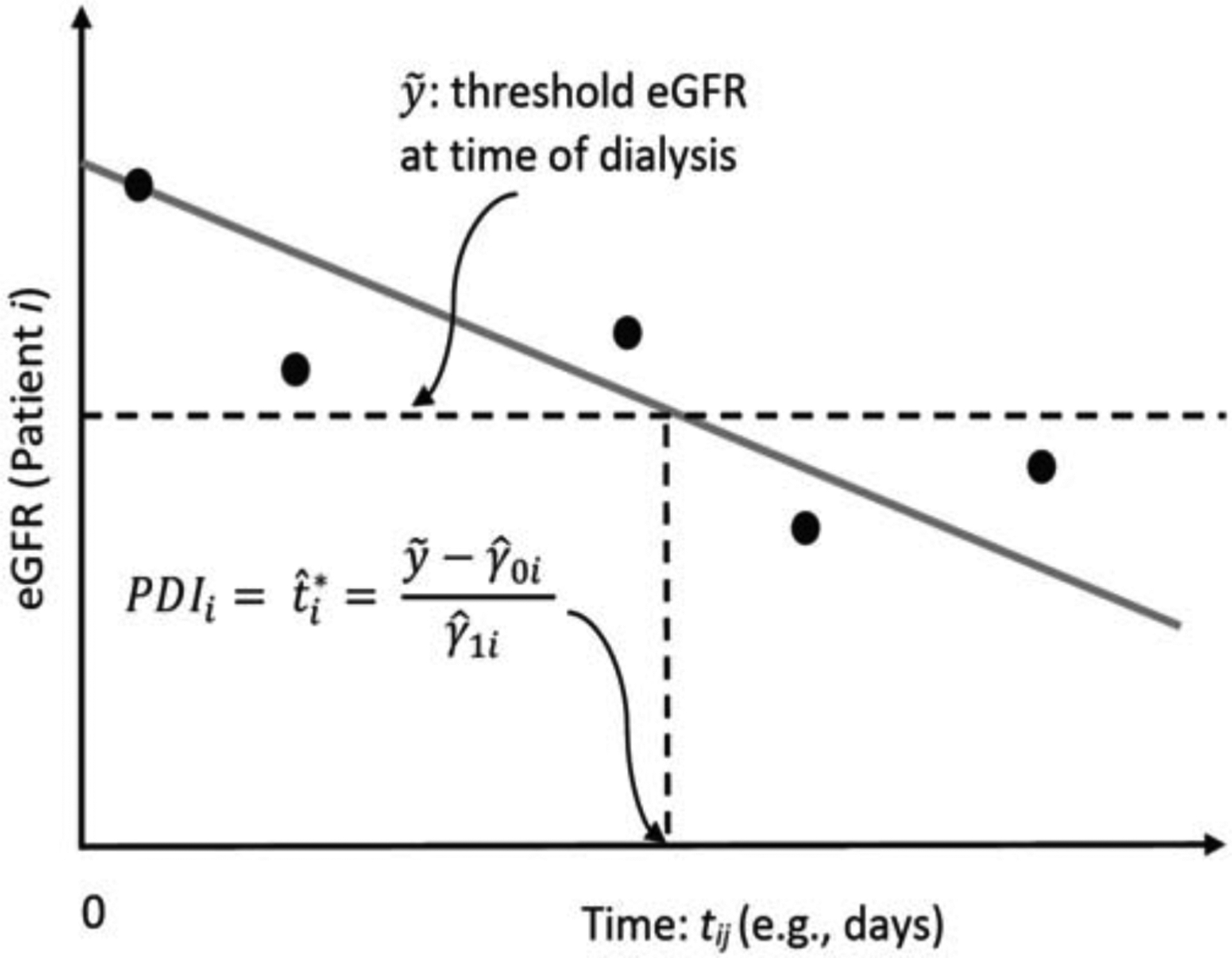
Estimation of putative dialysis initiation (PDI) time for a patient on conservative management via modeling of subject-specific longitudinal estimated glomerular filtration rate (eGFR) using linear regression (gray line). The estimated PDI time ${\hat{t}}_{i}^{*}$ is the time at which the expected eGFR level for patient ***i*** equals the threshold eGFR (e.g., the average eGFR level for patients who started dialysis).

To estimate the PDI time for a patient on CM Carson *et al*. (2009) applied linear regression to subject-specific longitudinal eGFR data. This is illustrated in Figure 2, where a linear regression model is fitted to five repeated eGFR measurements for subject *i*, with regression slope and intercept denoted by ${\hat{\gamma }}_{oi}$ and ${\hat{\gamma }}_{1i}$, respectively. The PDI time is then estimated as ${\hat{t}}_{i,LR}^{*}=\left(\tilde{y}-{\hat{\gamma }}_{oi}\right)/{\hat{\gamma }}_{1i}$, where $\tilde{y}$ is a threshold value of eGFR (kidney function level), such as the average eGFR value among those who initiated dialysis. Thus, as illustrated in Figure 2, if patient *i* on CM was to initiate dialysis, it is assumed that they would have initiated dialysis when their eGFR level is equal to the threshold $\tilde{y}$. When the threshold, $\tilde{y}$, is set to the average eGFR among patients in the study who did initiated dialysis, then the average eGFR level at the start of follow-up among patients on CM will not differ from the dialysis group.

Although it is feasible to fit a simple linear regression to subject-specific data to obtain an estimate of PDI time when number of eGFR measurements is greater than 2, the number of repeated measurements for studies with 2 to 4 years of follow-up typically have fewer than 15 eGFR measurements, for instance. Therefore, regression estimates, ${\hat{\gamma }}_{oi}$ and ${\hat{\gamma }}_{1i}$, can be unstable for many patients in a given dataset with low number of repeated eGFR measurements. An alternative which is more efficient and stable is to fit a linear mixed effects (LME) model to all patient data. More specifically, fit the model *Y*_*ij*_ = *β*_0*i*_ + *β*_1*i*_ + *e*_*ij*_ for *j* = 1, …, *n*_*i*_ measurements for subjects *i* = 1, …, *N*, where *β*_0*i*_ = *β*_0_ + *b*_0*i*_ and *β*_1*i*_ = *β*_1_ + *b*_1*i*_ are subject-specific intercept and slope, respectively, with random effects *b*_*i*_ = (*b*_0*i*_, *b*_1*i*_) ~ *N*(0, Σ) independent of measurement error *e*_*ij*_, and Σ is a 2×2 covariance matrix. The LME subject-specific estimate (best linear unbiased prediction), ${\hat{\beta }}_{0i}$ and ${\hat{\beta }}_{1i}$, can then be used to estimate the PDI for subject *i* as

$${\hat{t}}_{i,LME}^{*}=\frac{\tilde{y}-{\hat{\beta }}_{0i}}{{\hat{\beta }}_{1i}}=\frac{\tilde{y}-\left({\hat{\beta }}_{0}-{\hat{b}}_{0i}\right)}{\left({\hat{\beta }}_{1}-{\hat{b}}_{1i}\right)},$$

where $\tilde{y}$ is the threshold eGFR level described earlier.

### 2.2. Simulation Design and Model

To assess the efficacy of PDI time estimation, data (eGFR trajectories) were generated from the following LME model,

$$Y_{ij}=\beta_{0}+b_{0i}+\beta_{1}t_{ij}+b_{1i}t_{ij}+e_{ij}$$

with random effects *b*_*i*_ = (*b*_0*i*_, *b*_1*i*_) ~ *N*(0, Σ), $\Sigma =\left[\sigma_{1}^{2},\rho \sigma_{1}\sigma_{2};\rho \sigma_{1}\sigma_{2},\sigma_{2}^{2}\right]$, *σ*_1_ = 6.3, *σ*_1_ = 0.001, *ρ* = 0.6, $\sigma_{e}^{2}=4$, maximum follow-up time of about 5 years from baseline (time 0), and (*β*_0_, *β*_1_) = (25, −0.011). The relative parameter values for the model reflects our experience with eGFR trajectories of advanced CKD patients in practice, including baseline eGFR, typical declining *β*_1_ and positive covariance (and magnitude) between the random intercept and slope. Furthermore, the measurement time points, *t*_*ij*_, were generated to mimic a study where patients are recruited in the first two years and eGFR measurement commences after study entry and then longitudinal measurements taking place randomly at either 5, 6, or 7 months apart (which is not atypical). The average eGFR at baseline is 25 ml/min/1.73m^2^ and the regression coefficients and covariance matrix parameter values were chosen to mimic typical eGFR trajectories in advance CKD (stage 3b/moderate to severe: eGFR 30–45 and stage 4/severe loss of kidney function: eGFR 15–29 ml/min/1.73m^2^) [25] with baseline average eGFR of 25. We used the eGFR threshold $\tilde{y}=10\;\text{mL}/\text{min}/1.73{\text{m}}^{2}$, which is the average eGFR at initiation of kidney replacement therapy for incident patients in the United States in 2019 (latest USRDS data, N = 131,585)[1]. We compare the true and estimated PDI times (day of start of follow-up) for CM patients for 200 Monte Carlo datasets each of size 1,000 subjects using average mean absolute deviation. (Details are provided in Section 3.1.)

### 2.3. Simulation of Putative Survival Times

**Figure 3:**
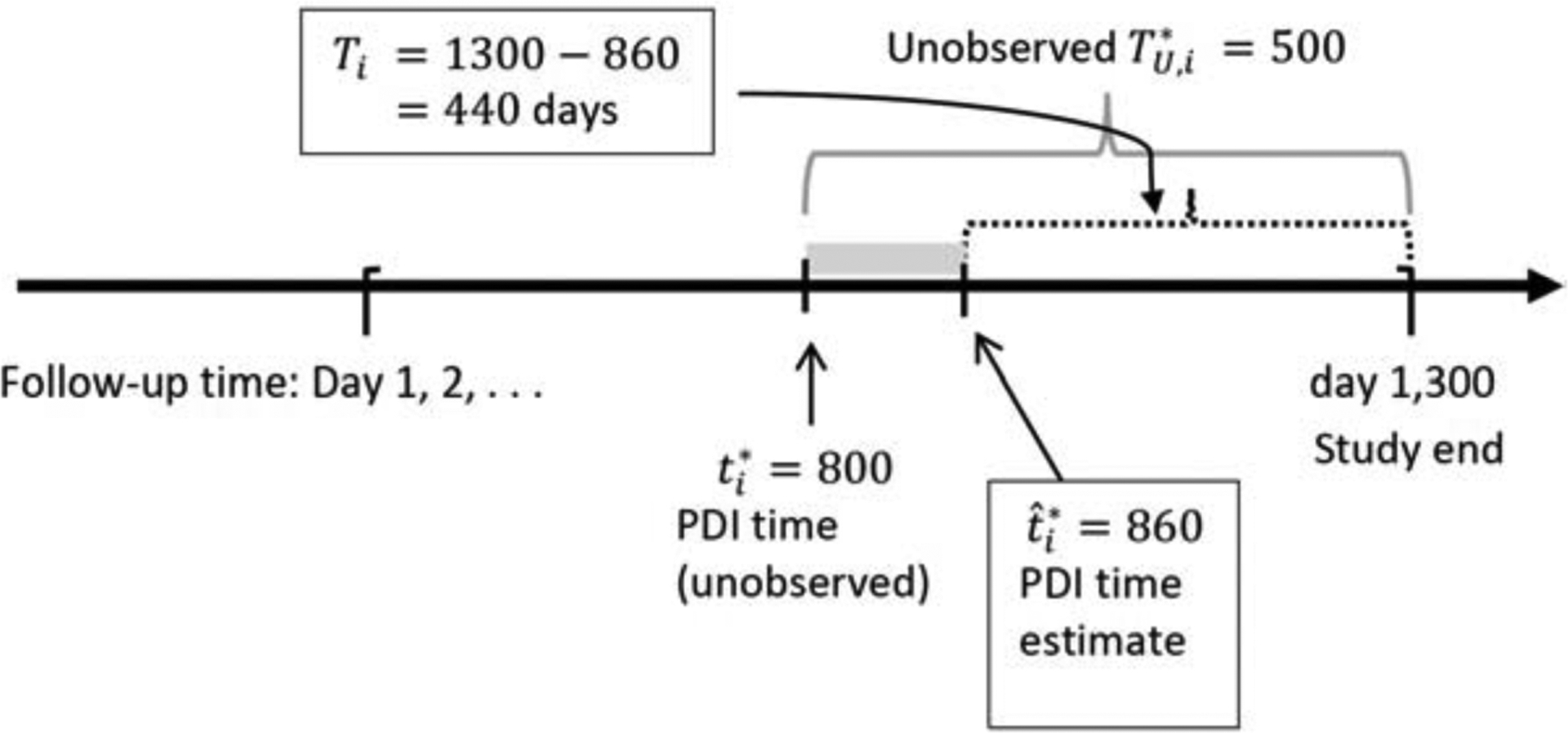
Generation of putative/estimated survival time for patients on conservative management: Illustrated is an unobserved survival time of 500 days (day 800 to the end of study on day 1,300) and the estimated (“observed”) survival time using the estimated putative dialysis initiation (PDI) on day 860 results in a “observed” survival of 440 days (day 860 to the end of the study).

Next, we consider estimation of the survival distribution based on estimated PDI time and compare that to the survival distribution based on the true PDI time. For the generation of survival times (and censored times) for patients in the CM group, denote the *unobserved* survival time for patient *i* as $T_{U,i}^{*}=\text{min}\;\left(T_{i}^{*},C_{i}\right)$, where $T_{i}^{*}\sim F_{T^{*}}\left(\theta_{1}\right)$ and $C_{i}^{*}\sim F_{C}\left(\theta_{2}\right)$ are the true survival and censoring times distributions with parameters *θ*_1_ and *θ*_2_, respectively. Let $\delta_{i}=I\left(T_{i}^{*}<C_{i}\right)$ be the censoring indicator where *I*(*A*) is the indicator function for event *A* and the observed survival time is $T_{i}=\text{min}\;\left(T_{i}^{*},C_{i}\right)$. However, for patients in the CM group the observed survival time *T*_*i*_ is “unobserved” because the start of follow-up time in unknown and needs to be estimated. Thus, to estimate *T*_*i*_ for a patient on CM, the follow-up time starts at the estimated PDI time, ${\hat{t}}_{i}^{*}$. Figure 3 illustrates an example where the study ends on day 1,300, the unobserved survival time $T_{U,i}^{*}=500$ days and, hence, the true (unknown) PDI time, *t*_*i*_, is day 800. If the estimated PDI time is day ${\hat{t}}_{i}^{*}=860$, then the estimated (“observed”) survival time is 440 days (from day 860 to the end of the study on day 1,300). For patients in the dialysis group, since the start of follow-up time is known, the observed survival time is simply $T_{i}=\text{min}\;(T_{i}^{*},C_{i})$. Survival and censoring times were generated from exponential distributions with density function $f(t)=\theta \;\text{exp}(-\theta t):T_{i}^{*}\sim Exp(0.14)$ and $C_{i}^{*}\sim Exp(0.08)$ with event rate of about 63%. Similarly, for Weibull distributed survival time, $T_{i}^{*}\sim \text{ Weibull }(2,1000)$ and *C*_*i*_ ~ Weibull(2, 1250), where Weibull(*a*, *b*) denotes the Weibull distribution shape parameter *a*, scale parameter *b*, and density function $f(t)=\text{exp}\left(\frac{a}{b}\right){\left(\frac{t}{b}\right)}^{a-1}\text{exp}\left\{-{\left(\frac{t}{b}\right)}^{a}\right\}$.

## 3. RESULTS

### 3.1. Estimation of PDI times

**Figure 4:**
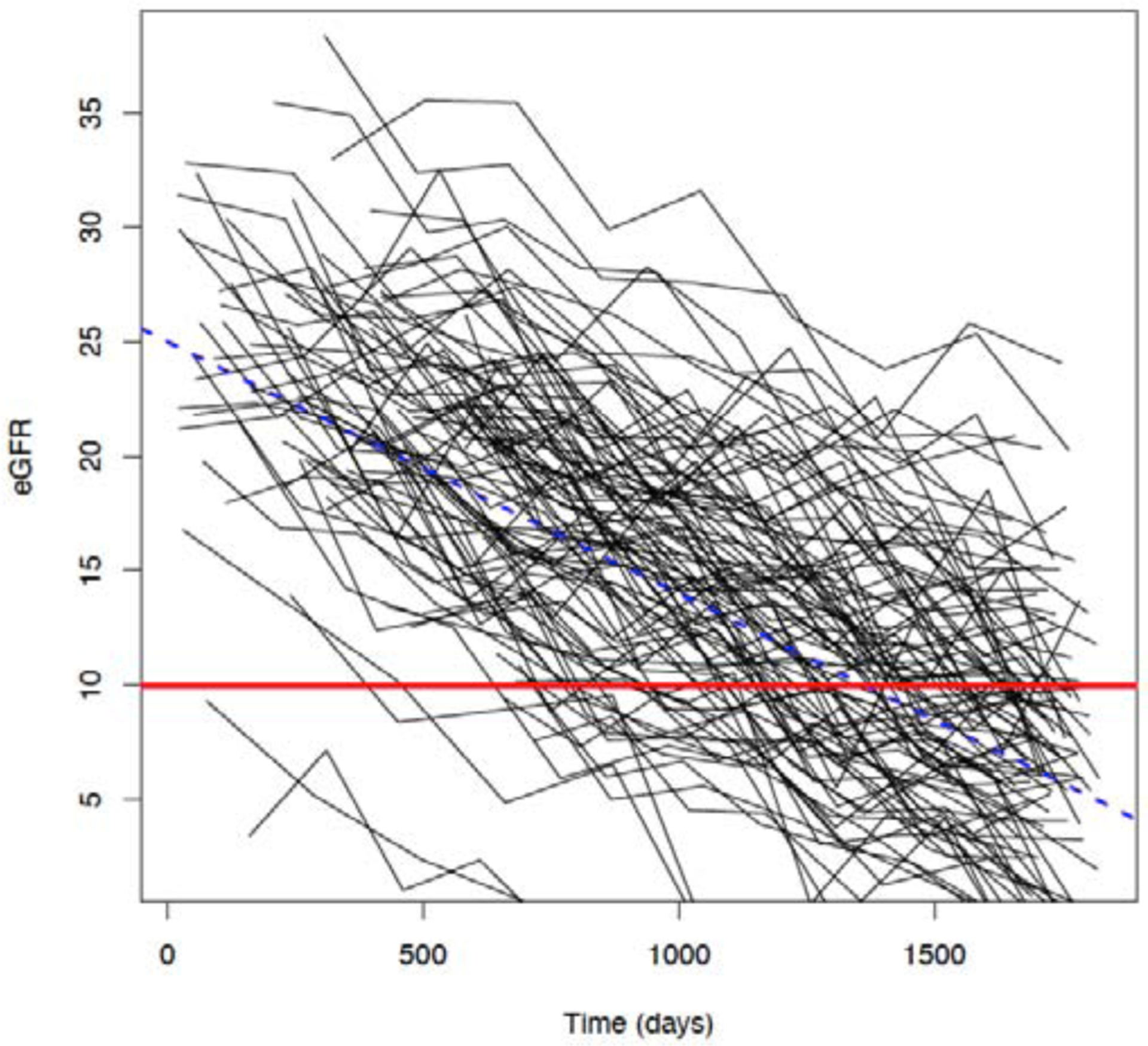
Randomly selected 100 subjects from a simulated dataset of estimated glomerular filtration rate (eGFR) trajectories over time (5 years/1825 days): Shown are 100 eGFR trajectories along with eGFR threshold 10 mL/min/1.73m^2^ (red line) and true average eGFR = 25 – 0.011× time.

Figure 4 displays eGFR trajectories over time (5 years, 1825 days) for 100 randomly selected subjects from a typical simulated dataset of 1,000 subjects (Section 2.2), along with eGFR threshold 10 mL/min/1.73m^2^ (red horizontal line) and true average/expected eGFR = 25 – 0.011× time (*t*_*ij*_). The average (over 200 simulated datasets) of the minimum, median and maximum number of observations per subject was 6, 7.5, and 13, respectively.

For each dataset, we calculated the mean absolute difference/deviation (MAD) between the true $\left(t_{i}^{*}\right)$ and estimated (${\hat{t}}_{i,LR}^{*}$ and ${\hat{t}}_{i,LME}^{*}$), $\text{PDI time: }\sum_{i=1}^{N}\left|t_{i}^{*}-{\hat{t}}_{i,**}^{*}\right|/N$, *N* = 1000, where ** denotes LR or LME. Summary of the mean absolute difference over 200 Monte Carlo datasets for estimation of putative dialysis initiation (PDI) time using the LME model and individual linear regression (based on subject-specific data, i.e., LR) are summarized in Table 1. The average MAD for LME PDI estimate was 83.5 (SD 4.0) compared to LR PDI average MAD of 115 (SD 9.6). Thus, the average error is higher for LR as well as more variable (due to the small sample size because only data from subject *i* is used). Not surprisingly, the performance of LR further deteriorates when the number of observations per subject is further reduced (not shown).

### 3.2. Estimation of Survival Based on PDI Times

**Figure 5:**
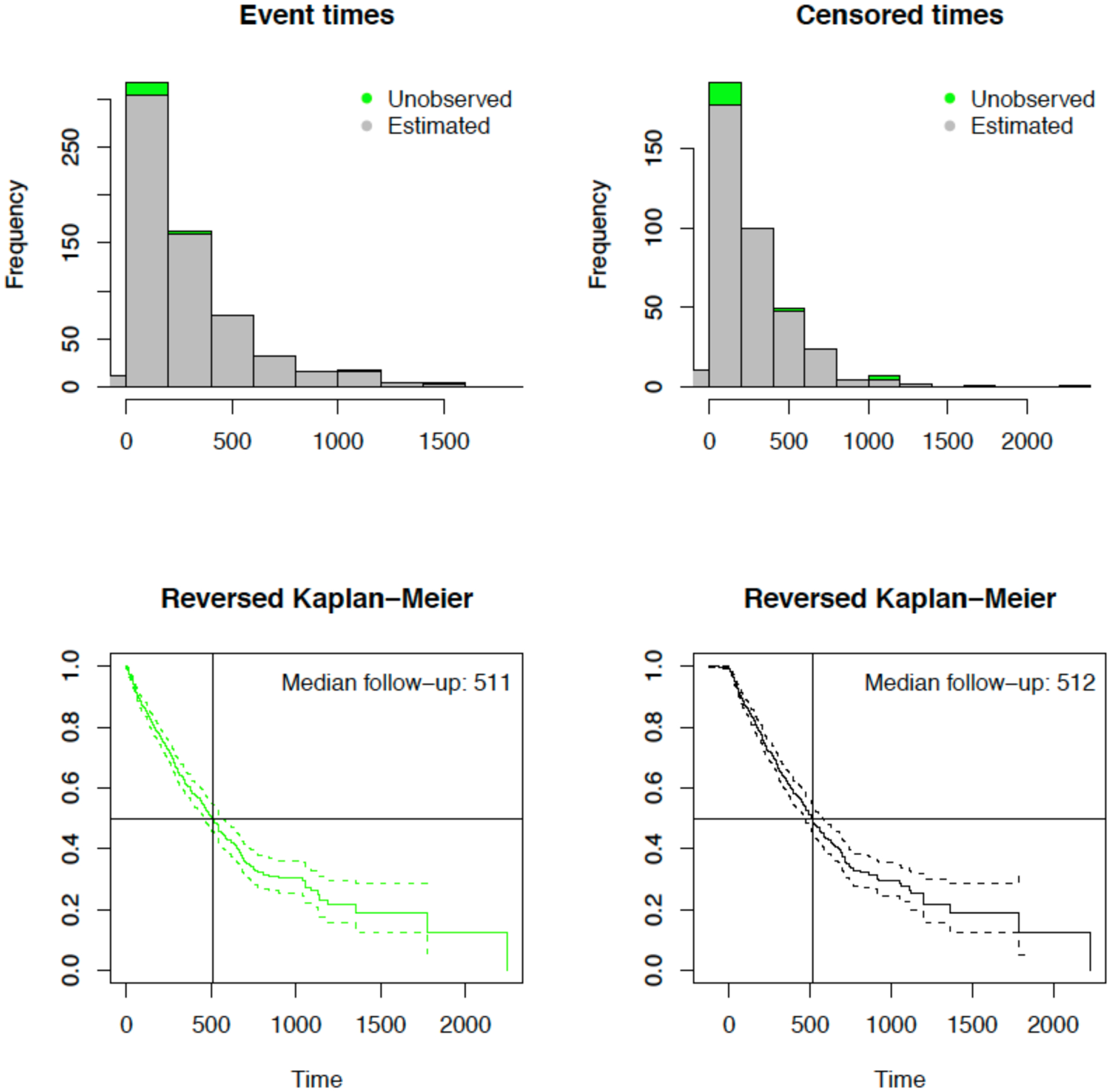
Characteristics of simulated exponential survival time: (top row) Distribution of survival (event and censored) times for true/unobserved (green) and from estimated PDI time (gray) and (bottom row) distribution of follow-up times for true and from estimated PDI time using reverse Kaplan-Meier.

We examined the efficacy of estimating the survival distribution for patients on CM when the true PDI times are unknown/unobserved and, therefore, must be estimated using LME model. As detailed in Section 2.3, the “observed”/estimated survival time is based on the starting the follow-up time at the estimated PDI time, ${\hat{t}}_{i}^{*}$, for patient *i*. For the first case of exponential survival time, Figure 5 summarizes the basic characteristics of simulated exponential survival time based on true true/unobserved (green) survival times and from estimated survival times via PDI follow-up time (gray). Median follow-up time, based on reverse Kaplan-Meier (KM) curve, was similar between true survival time (median 511) compared to estimated follow-up time based on PDI (median 512); see Figure 5 for details. Characteristics were similar for the second case of Weibull distributed survival times (not shown).

**Figure 6:**
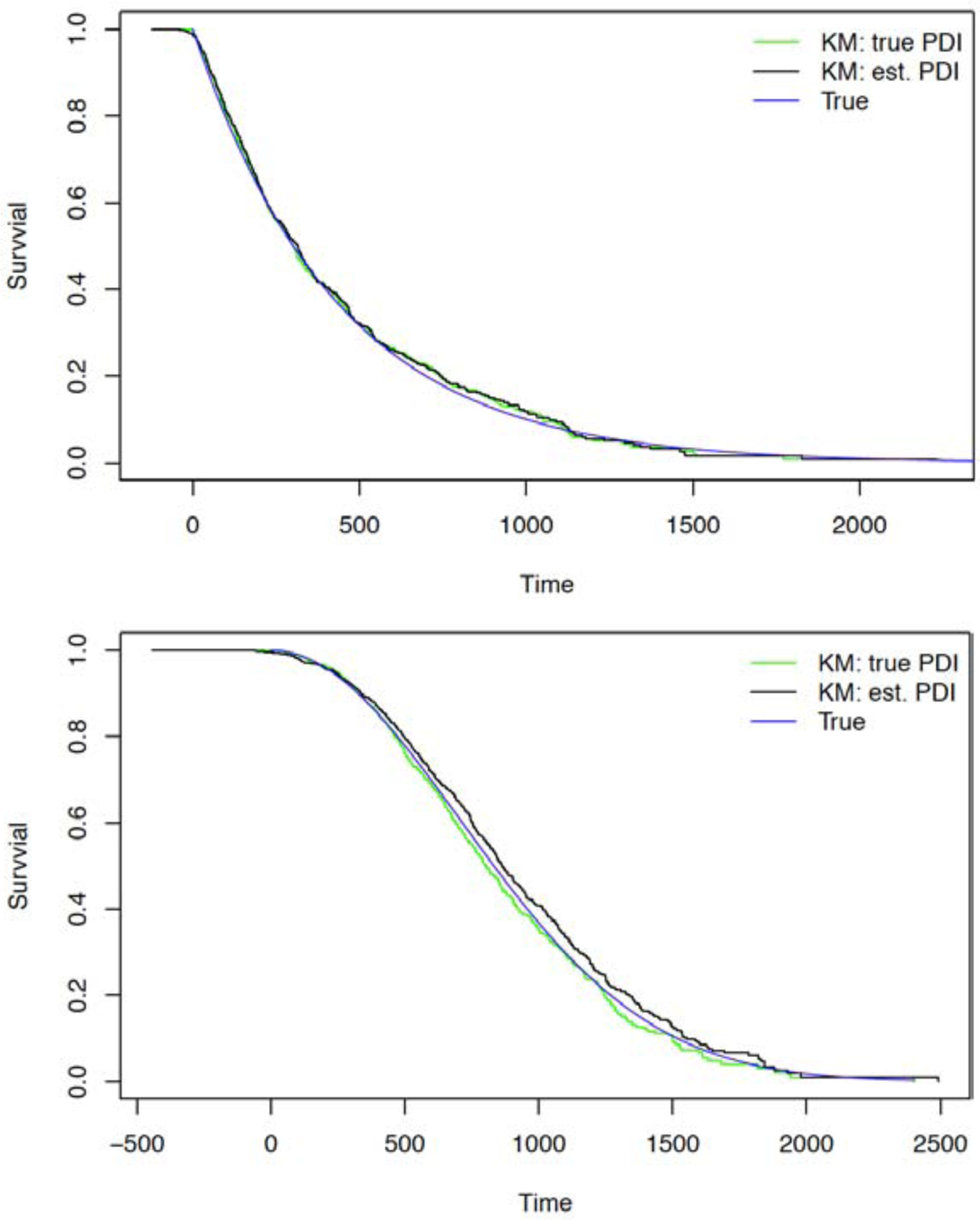
Estimation of survival distribution using putative dialysis initiation (PDI) time: True survival distribution (blue), along with Kaplan-Meier (KM) curves estimates based on the unobserved survival time (unobserved PDI, ***t***_***i***_; green curve) and the estimated survival time (based on estimated PDI, ${\hat{t}}_{i}^{*}$; black curve); exponential (top) and Weibull (bottom) survival times.

Typical estimation of the survival distribution is illustrated in Figure 6, which displays the true survival distribution (blue), along with KM estimates based on the unobserved survival time (unobserved PDI, *t*_*i*_; green curve) and the estimated survival time (based on estimated PDI, ${\hat{t}}_{i}^{*}$; black curve). Survival distribution estimation based on estimated PDI tracks the unobservable PDI well and both targets the true survival distribution. Effective estimation of survival based on estimated PDI follow-up was similar for exponential and Weibull distributed survival times.

## 4. CONCLUSION AND DISCUSSION

In this work we examined the validity of the use of putative dialysis initiation time to determine the start of follow-up for longitudinal (retrospective and prospective) cohort studies of advanced CKD patients choosing conservative management where the start of follow-up technically does not exist since patients on CM do not initiate dialysis. Thus, PDI time or the time at which a patient on CM would have started dialysis, estimated based on their kidney function decline (eGFR) is a useful concept. We proposed a stable estimate of PDI time based on LME modeling of the eGFR trajectories and showed that it targets the true PDI time. Furthermore, we illustrated via simulation studies that the survival distribution based on estimated PDI time also targets the underlying true survival distribution.

We note that the proposed approach to estimation of PDI time effectively “matches” the average eGFR (kidney function level) between exposure groups (CM and dialysis), in addition to providing a stable subject-specific start of follow-up time (PDI time) for patients on CM. However, depending on the analytical/scientific objective, it may also be necessary to account for the effects of other covariates. For instance, to compare survival between CM and dialysis groups, it is also important to account for relevant demographic, social-economic and laboratory measures including eGFR, as well as comorbidities and medication. This can be achieved through propensity score [28] matching, followed by estimation of PDI times and comparison of survival via Kaplan-Meier of the matched (CM and dialysis) cohorts. Alternatively, multivariate Cox regression can be used to adjust for confounders and with the estimated PDI times as the start of follow-up times for patients in the CM group.

We also note that the aforementioned approach to use propensity score matching combined with estimated PDI time for patients on CM aims to mimic the context of a randomized trial (with balanced baseline factors, including eGFR) and well-defined at-risk time. This is needed to provide valid comparative analysis of outcomes between exposure groups. Additionally, an important issue in comparing outcomes requiring follow-up time with respect to CM and dialysis is immortal time bias [26–27], where, by definition, dialysis patients cannot die prior to dialysis. Also, overall survival starting from a defined study entry time point (see “start of study” mark in Figure 1), may of interest in some situations. In such cases, analytic approaches using time-varying (time-dependent) treatment variable, e.g., in a Cox model for survival outcome, may be appropriate.

Finally, we note that although the current problem shares some similarities to other problems where the time-dependent propensity score matching approach [29] has been used, there are distinct differences. More specifically, Lenain *et al*. (2021) [30] applied this approach to emulate a (conceptual) clinical trial (with time-dependent exposure) examining overall survival in patients with *kidney failure* between patients “randomized” to transplantation versus transplant “waitlist,” among patients *eligible* for kidney transplantation. Although on the surface there are technical similarities, there are also distinct differences in the context of conservative management of CKD patients. First, for patients on dialysis awaiting kidney transplantation, their kidney functions have failed (and they are on dialysis) whereas in the current context, we are monitoring the kidney function trajectories (eGFR) of CKD patients on CM to estimate their putative dialysis transition time (if they choose to start dialysis). Thus, the key information used, which is the use of a patient’s (remaining) kidney function to determine a potential time of dialysis treatment, is not available (and not relevant) in longitudinal studies of ESKD patients, including patients with kidney failure on a kidney transplant waitlist. A second important distinction is that patients on a kidney transplant waitlist are receiving the “same” treatment, namely dialysis, whereas in the current context, there is no uniform treatment [31] prior to the start of follow-up. Another important distinction is that it is natural to view the time of kidney transplant as a time-dependent treatment as was done in Lenain *et al*. (2021) since (a) the start of the baseline period (when patients were added to the transplant waitlist) is well-defined and (b) treatment is fairly uniform (all patients on dialysis at the time of waitlist) and the interest is on overall survival. This is similar to the goal of comparing overall survival as depicted in Figure 1 starting at the “start of study” time mark (e.g., eGFR ≤ 25). However, when the interest is on “post-dialysis” survival, as is the focus of this paper, estimating the putative dialysis transition time is a useful concept.

## Figures and Tables

**Table 1:** Mean Absolute Deviations for Estimation of Putative Dialysis Initiation (PDI) Time using Linear Mixed Effects (LME) Model and Individual Linear Regression (LR) using Subject-Specific Data; Results are from 200 Monte Carlo Datasets

Median absolute deviation	Min	Q1	Median	Mean	Q3	Max	SD
PDI estimate: LME	75.7	80.9	83.5	83.6	86.1	97.1	4.0
PDI estimate: LR	101.5	111.0	114.0	115.0	117.0	221.1	9.6

Q1: first quartile, Q3: third quartile, SD: standard deviation.

## LIST OF ABBREVIATIONS

CDC: Center for Disease Control and Prevention
CKD: chronic kidney disease
CM: conservative management
eGFR: estimated glomerular filtration rate
ESRD: End-Stage Renal Disease
ESKD: End-Stage Kidney Disease
HRQOL: health-related quality of life
KM: Kaplan-Meier
LME: linear mixed effects
LR: linear regression
MAD: mean absolute deviation
NIDDK: National Institute of Diabetes and Digestive and Kidney Diseases
PDI: putative dialysis initiation
USRDS: United States Renal Data Systems
